# Intergenerational trauma: A silent contributor to mental health deterioration in Afghanistan

**DOI:** 10.1002/brb3.2905

**Published:** 2023-02-27

**Authors:** Hamid Ullah, Hafsa Ahmad, Zoaib Habib Tharwani, Sean Kaisser Shaeen, Zainab Syyeda Rahmat, Mohammad Yasir Essar

**Affiliations:** ^1^ Faculty of Medicine Dow University of Health Sciences Karachi Pakistan; ^2^ Department of Dentistry Kabul University of Medical Sciences Kabul Afghanistan

**Keywords:** conflict, humanitarian crisis, intergenerational trauma, mental health, poverty, Post Traumatic Stress Disorder, trauma, violence

## Abstract

Multiple theories, including family systems, epigenetics, attachments, and many others, have proposed mechanisms for trauma transmission from generation to generation. Intergenerational trauma is today one of the most important psychosocial issues affecting Afghans’ mental health and psychology, with the potential to affect subsequent generations. A variety of factors have impacted the mental health of the Afghan population over the years, including years of conflict, socioeconomic instability, natural disasters, chronic drought conditions, economic turmoil, and food insecurity, all of which have been exacerbated by recent political turbulence and the The Coronavirus pandemic COVID‐19 pandemic that has further increased the susceptibility to intergenerational trauma among the Afghan population. International bodies must play a role in addressing intergenerational trauma among Afghans. Breaking the chain in future generations will be possible by resolving political issues, providing adequate health facilities, financial support, and eliminating stigmas associated with mental health issues.

## Graphical abstract

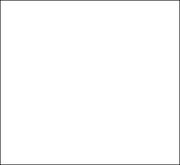

## 1 INTRODUCTION

Intergenerational trauma, also known as transgenerational trauma, is an emotional or psychological trauma experienced by a group of people that affects the health and well‐being of individuals in successive generations (Cerdeña et al., 2021). The impacted generations will have signs and symptoms of depersonalization, emotional numbness, depression and anxiety, blemished life skills, a lack of self‐worth, and post‐traumatic stress disorder (Raypole, 2022). The triggering traumas can be personal, such as intimate partner violence, or collective, as in war or genocide. Intergenerational trauma is passed down in complex and subtle ways through attachment relationships and within family and community groups (Isobel et al., 2020). One study discovered that successors of Holocaust survivors had shown changes in stress hormonal changes, indicating a change in their genetic makeup, implying that stressful experiences experienced by parents and grandparents can affect children even before they are born. Even after the threat has passed and they have resettled in a safe country, the body does not return to an un‐stressed state, and this response causes physical and psychological problems for future generations (A silent mental health crisis plagues Afghans at home & abroad, 2022; Sigal et al., 1988).

Afghans have suffered from decades of conflict, displacement, poverty, and unemployment. According to a 2018 EU survey, 85% of Afghans had witnessed at least one traumatic event. According to the Afghanistan Ministry of Public Health, one in every two Afghans suffers from psychological distress, and one‐fifth of the population has difficulty performing routine tasks (A silent mental health crisis plagues Afghans at home & abroad, 2022). Intergenerational trauma, particularly among the Afghan population, has had a significant impact on children, grandchildren, and subsequent generations by increasing susceptibility to poor physical and mental health and psychosocial problem (What about the mental health of Afghan children?, 2022).

It is reported that 50% population in Afghanistan suffers from psychological distress and impairment due to mental health issues (Kovess‐Masfety et al., 2021). Afghanistan is described as a trauma state, with over 70% of Afghans requiring mental health support (ReliefWeb, 2022). Several factors have proven to be responsible for disrupting the public's approach to seeking mental health services. This article will examine evidence‐based interventions and strategies to support succeeding generations affected by intergenerational trauma and its health implications among the Afghan population.

## 2 CHALLENGES

A multitude of factors, including social, political, and economic factors, have affected the mental health of Afghans over the years, and the recent humanitarian crises along with the advent of COVID‐19 have further increased the suffering as well as the susceptibility to intergenerational trauma among multiple Afghan families (Cardozo et al., 2004).

### 2.1 Effects of war and conflicts on mental health

People in Afghanistan have been experiencing a continuous state of violence and conflict for over two decades, which has led to the buildup of serious mental health issues among Afghans (Mohd Saleem et al., 2021). These mental health issues are more pronounced among individuals who were exposed to traumatic events such as witnessing violence, bombarding, the death of family members, mass killings, and living in a war zone (Panter‐Brick et al., 2009).

### 2.2 Effect of regime change on mental health and intergenerational trauma

The recent change in power has increased emotional and mental strain on Afghans after the Taliban took control of Kabul on August 15, 2021 (The Indian Express, 2021) (Afghanistan's Silent Mental Health Crisis | Human Rights Watch, 2023). The annexation of Afghanistan by the Taliban has augmented the already present humanitarian crisis. According to The United Nations Assistance Mission in Afghanistan UNAMA's report, 876 civilians lost their lives, and 1915 civilians were injured between January and April 2021. Of serious concern is the fact that women and children make up 46%, almost half, of all civilian casualties (UNAMA., 2021) (Cardozo et al., 2004). Extremely traumatic events such as these cause drastic effects on the mental health of the population and can potentially impact the mental health of subsequent generations (Lombardi et al., 2020).

### 2.3 Effect of the COVID‐19 pandemic on mental health

The COVID‐19 pandemic emerged as a serious healthcare challenge to Afghanistan and added to the already present humanitarian crisis. The first case of COVID‐19 was reported on February 24, 2020, in Herat, Afghanistan, and since then COVID‐19 has become a healthcare emergency in the country (Shah et al., 2020). According to WHO, from January 3, 2020, to May 16, 2022, there have been 179,267 confirmed cases and 7690 deaths due to COVID‐19 in Afghanistan (Afghanistan: WHO Coronavirus Disease (COVID‐19) Dashboard With Vaccination Data, 2022). The widespread pandemic has caused the deterioration of an already fragile healthcare system. The recurrent lockdown has resulted in significant psychological effects on mental health outcomes (Talevi et al., 2020). Moreover, an important contributor to this state of psychological distress among Afghans is the lack of psychiatrists, inadequate mental health facilities, and social stigmas impeding the public's approach to seeking mental health services, which can further aggravate the prevalence of intergenerational trauma among the Afghan population (Human Rights Watch, 2023).

### 2.4 Socio‐economic instability and issues of Internally Displaced People (IDPs)

Ongoing political conflicts, the COVID‐19 pandemic, economic turmoil, and chronic drought conditions have all collectively escalated the food insecurity crisis in Afghanistan (The World Bank, 2021)(Islam et al., 2022). More than half of Afghanistan's 30 million population lives below the poverty line, and 11 million are acutely and severely food insecure (U.S. Agency for International Development, 2020) (Isobel et al., 2020). Food insecurity and poverty are specifically associated with mental health issues, anxiety, and psychological distress among people. As a result, Conflict‐induced displacement has become a common coping strategy for many Afghans over multiple generations (IDMC, 2020). (Mohd Saleem et al., 2021). The mental health consequences in the displaced population and refugees are more pronounced due to traumatic exposure before or during migration making them more vulnerable to intergenerational trauma (ReliefWeb., 2012).

### 2.5 Efforts and recommendations

Various factors are working simultaneously to address intergenerational trauma, which holds a significant influence over the general population. These factors also work from different angles to accomplish this, with some focusing on environmental aspects as well as other types of contributing factors that lead to an increase in mental health cases. This is done while simultaneously working toward treating mental health issues directly in some form or another. For example, The United Nations International Children's Emergency Fund (UNICEF) supports Afghan civilians by providing therapeutic food in Afghanistan, which has saved several lives each month (Qayoumi, 2022). As studies have shown that food insecurity has been associated with mental illness, therefore, not only does the therapeutic food save physical lives but it also benefits mental health as well (Fang et al., 2021).

Along with improving the environment, some organizations are implementing strategies to treat mental health issues present in the population. The Borgen Project (2020) has worked to provide crucial mental health services by providing healthcare facilities and training for healthcare workers in which they can identify mental health disorders with the ability to create corresponding treatments for them (Shah et al., 2020). The Tabish Social health education organization is a local organization that has worked with national and internationally certified psychotherapists and counselors to provide counseling services in its clinics or even in remote areas by deploying the same individuals to the field. This organization also made its work public by working with more than 100 local radio stations across the country to provide information about its services and programs for the general population (Sigal et al., 1988). In addition to the radio stations, there are over 60 operational hotlines in Afghanistan that provide mental health support and offer referrals to specialized services geared toward addressing their mental health concerns (Islam et al., 2022). Recommendations for parties interested in improving the various issues contributing to the mental health condition of Afghans can start with providing basic needs for the general population such as clean food and water. With Afghanistan suffering a drought and increased poverty rates, an increasingly larger number of individuals are suffering from conditions such as malnutrition, as the United Nations reported acute hunger in the country has risen from 14 million in July to 23 million in March with an astonishing 95% of Afghans not getting enough to eat (UN News, 2022).

Another important strategy to improve the population's mental health is providing funds for the enervated healthcare system. Due to recent political turmoil, many international donors have cut funding significantly or even together (HealthNet TPO, 2021). Although the Taliban have made strides to appeal to them for the betterment of its country, and some donors have started providing financial support once again, there are still many who are hesitant. This hesitancy has caused many facilities to run short on medical supplies and staff as the current funding does not suffice (Forbes, 2022). Therefore, these donors should be urged to donate aid to avoid famine across the country. Last, a stigma surrounding the idea of mental health remains prominent in Afghanistan and prevents many people from seeking help. To reduce the stigma, those in the medical field should work in tandem with social media influencers, radio hosts, the Taliban, and religious leaders to educate the citizens on the need and benefits of seeking help for mental health issues.

## 3 CONCLUSION

A decades‐long conflict, humanitarian crisis, a collapsing economy, war, and political violence, and the COVID‐19 pandemic have all impacted the mental health, attitude, and behavioral patterns of Afghans. All these factors also affect communal productivity, resulting in long‐term mental health consequences and catastrophic outcomes for the masses spans generations. Various NGOs must improve access to and quality of mental health services, as well as train psychosocial counselors, doctors, midwives, and nurses in the fundamentals of mental health support, facilitating them to provide support to community members and patients within health facilities. As a result, immediate steps should be taken to mitigate the physical, psychological, and behavioral long‐term consequences of intergenerational trauma. The authorities must address this issue by providing financial assistance to alleviate poverty and food insecurity, as well as allocating adequate funds to health sectors and establishing mental health centers across the country. To address intergenerational trauma‐related mental health issues, different sectors must raise public awareness about physical and mental health issues.

## AUTHOR CONTRIBUTIONS

Hamidullah wrote the abstract, introduction, and conclusion and edited the revised draft. Hafsa wrote the challenges. Sean Kaisser Shaeen wrote the efforts and future recommendations. Mohammad Yasir Essar, Zoaib Habib Tharwani, and Zainab Syyeda Rahmat made the critical comments and revisions. All authors revised and approved the final draft.

## CONFLICT OF INTEREST STATEMENT

The authors declare that they have no competing interests.

### PEER REVIEW

The peer review history for this article is available at https://publons.com/publon/10.1002/brb3.2905.

## Data Availability

N/A
